# Quantifying plant hydraulic function becomes a tall order

**DOI:** 10.1093/jxb/eraa240

**Published:** 2020-07-06

**Authors:** Robert P Skelton

**Affiliations:** South African Environmental Observation Network, Fynbos Node, Newlands, Cape Town, South Africa

**Keywords:** Hydraulic conductance, plant form and function, plant hydraulics, water transport, xylem embolism

## Abstract

This article comments on:

**Soriano D, Echeverría A, Anfodillo T, Rosell JA, Olson ME**. 2020. Hydraulic traits vary following tip-to-base conduit widening in vascular plants. Journal of Experimental Botany **71**, 4232–4242.

**In this issue, Soriano *et al*. (2020) present a thought-provoking study that investigates whether a well-described aspect of plant form (the scaling of conduit size with plant height) may cause coordinated changes in other hydraulic traits. In doing so, the authors build on a long and rich history of studies investigating the relationship between plant architecture and hydraulic function (Zimmermann, 1983; Tyree and Sperry, 1989; Choat *et al.*, 2005), and reveal that quantifying plant hydraulic function might be a taller order than suspected.**

## Scaling of form and function with plant height

According to the pipe model of fluid transport, the resistance to flow through a capillary increases linearly with its length and decreases in proportion to the diameter raised to the fourth power. Although this model is an oversimplification of flow in plants (see below), it provides an entry point to understanding variation in plant anatomy with height. In woody plants, tip-to-base conduit widening is expected to maintain water transport efficiency per unit leaf area constant as plants grow taller.

Soriano *et al.* (2020) investigated whether the scaling of conduit size with plant height may also cause coordinated changes in other hydraulic traits, including the capacity of the xylem to withstand air entry which causes blockages (see Box 1). To do so, the authors compared anatomical and hydraulic measurements made on nursery-grown plants of different heights. Measurements were made on three woody tree species (two angiosperms and a conifer) and included vessel diameter, segment-specific hydraulic resistance, the ratio of sapwood area to leaf area (known as the Huber value), and xylem vulnerability to embolism.

**Box 1. Two mechanisms of embolism**

**Air-seeding:** as plants dehydrate, increasing tension (i.e. negative pressure) in the water column can cause bubbles of air to be drawn into the xylem from neighbouring conduits and/or expand (if already present in the sap). According to the air-seeding hypothesis, the capacity to withstand air entry and propagation through the network of xylem vessels or tracheids will be determined by the dimensions of the pit pore membranes of a vessel and the torus and margo of a tracheid.

**Freeze–thaw embolism:** when sap freezes, dissolved gases are forced out of solution to form bubbles in the ice. Upon thawing, these bubbles either dissolve back into the sap or expand if the sap is under tension. If the tension is great enough, the trapped air can expand to obstruct the entire xylem conduit (i.e. causing it to cavitate). Whether a bubble dissolves or expands depends on its internal pressure, which is a function of its radius of curvature, the surface tension of the xylem sap, and the xylem sap pressure. Susceptibility to freezing-induced cavitation should increase for greater tension (i.e. drought stress) and larger radius of curvature of bubbles. The latter will depend at least in part on the size (and volume) of the xylem conduit, because larger volumes of sap trap more air that can be frozen out of solution, forming larger bubbles.

Their results show that architectural or anatomical traits (i.e. vessel diameter, vessel number, and Huber value) co-vary with stem height, as expected. However, the authors also show that plant function changes with plant height: segment-specific water transport efficiency decreases and capacity to withstand xylem air entry increases with height. The authors conclude that future comparisons of plant function need to standardize for plant height. This finding is potentially significant for scaling up from plant trait to ecosystem behaviour because current models often do not incorporate height-related variation in plant capacity to withstand xylem embolism. Gathering such data with current techniques is both time-consuming and notoriously difficult.

## Inextricable yet complex link between form and function

**Fig. 1.**
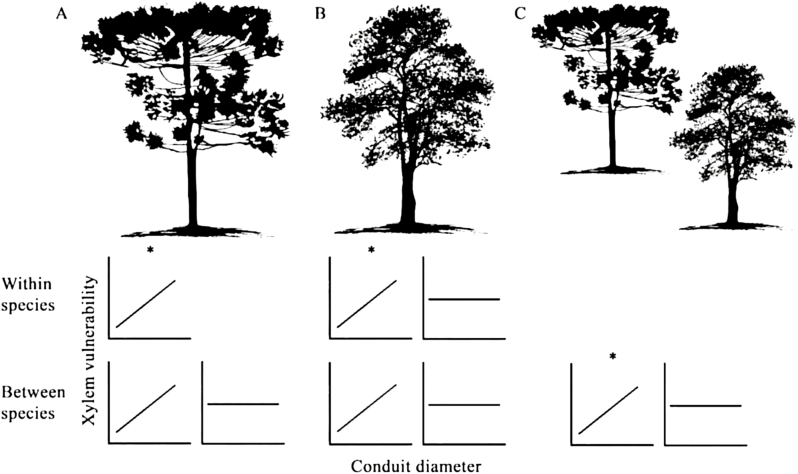
Decades of studies reveal no consistent relationship between xylem conduit diameter and xylem vulnerability to drought-induced embolism in major land plant groups. Studies investigating conifers (A), angiosperms (B), or mixtures of both major groups (C) frequently report either weak positive relationships or no relationship between these variables. Stronger relationships are often (but not always) observed in within- (top row) versus between-species comparisons (bottom row). The asterisks indicate patterns observed in Soriano *et al.* (2020). The text provides reference studies.

Plant biologists have been preoccupied with quantifying the intricate and complex links between xylem function and anatomy for centuries, and delving into this rich history reveals why the pursuit continues to remain relevant. Studies conducted over the past four decades demonstrate that there is no clear, consistent relationship between conduit diameter and xylem vulnerability to drought-induced embolism in major land plant lineages (Fig. 1). A weak positive or no relationship between conduit diameter and vulnerability to drought-induced embolism has been reported among conifers (e.g. Tyree *et al.*, 1994; Pittermann *et al.*, 2006; but see also Larter *et al.*, 2017; Fig. 1A), angiosperms (e.g. Hacke *et al.*, 2005; Cochard *et al.*, 1999; but see also Martinez-Vilalta et al., 2002; Fig. 1B), and mixed compositions of conifer and angiosperm species (e.g. Tyree and Dixon, 1986; Davis *et al.*, 1999; Fig. 1C). A lack of a relationship has been reported within angiosperm species (e.g. Cochard *et al.*, 1999; Choat *et al.*, 2005; but see also Hargrave *et al.*, 1994), but not in conifers. Thus, it is still true (two and a half decades after Tyree *et al*. reviewed the available data and first proclaimed it) that ‘a physiologist cannot predict the vulnerability of a species by measuring the mean conduit diameter’ (Tyree *et al.*, 1994).

However, if conduit diameter does not directly determine capacity to withstand xylem air entry during dehydration, why do we frequently observe relationships between these variables at all? By demonstrating that both variables might be associated with plant height, Soriano *et al.* (2020) suggest a possible explanation: conduit diameter and xylem vulnerability to dehydration-induced embolism are potentially linked to plant height via properties of pit pore membranes (the valves that connect adjacent xylem vessels in angiosperms or tracheids in conifers). Because efficiency of transport is related to both the conduit diameter and the properties of pores (in general thinner pore membranes allow water to pass more easily), taller trees might have to possess both wider conduits and larger or thinner pores. Thinner pore membranes may also make xylem conduits less safe (i.e. less capable of withstanding air entry and propagation). Flying close to the sun might be risky for trees too!

An alternative explanation is that conduit diameter and capacity to withstand xylem air entry and propagation could also be indirectly, but positively, related by independent associations with conduit volume or total wall area. Vessel diameter and vessel length are positively correlated (i.e. larger diameter vessels tend to be longer), meaning that vessel diameter will be linked to volume. In addition, larger volumes of sap tend to trap more air frozen out of solution (see ‘Freeze–thaw embolism’, Box 1), forming larger bubbles, which tend to expand more easily if the xylem thaws and is under tension. The area of overlap between adjacent vessels or tracheids might also be larger in larger diameter vessels. Extensive intervessel pitting in large vessels of trees could render these vessels more vulnerable to air entry. Exploring which of these hypotheses best explains plant capacity to withstand dehydration will be a priority for future studies to improve our understanding of drought tolerance. Thus, by demonstrating some of the complexity of plant form and function, Soriano *et al*. highlight that we still have much to learn about plant form and function. Although xylem vulnerability to dehydration-induced embolism might vary with height, we are far from having a full understanding of the causes of this. What remains clear is that the pursuit to reconcile plant anatomy and form with plant function will go on for a while yet.
